# Neural Coding of Movement Direction in the Healthy Human Brain

**DOI:** 10.1371/journal.pone.0013330

**Published:** 2010-10-13

**Authors:** Christopher D. Cowper-Smith, Esther Y. Y. Lau, Carl A. Helmick, Gail A. Eskes, David A. Westwood

**Affiliations:** 1 Department of Psychology, Dalhousie University, Halifax, Nova Scotia, Canada; 2 Department of Psychology, The University of Hong Kong, Hong Kong, China; 3 Department of Psychiatry, Dalhousie University, Halifax, Nova Scotia, Canada; 4 School of Health and Human Performance, Dalhousie University, Halifax, Nova Scotia, Canada; The University of Western Ontario, Canada

## Abstract

Neurophysiological studies in monkeys show that activity of neurons in primary cortex (M1), pre-motor cortex (PMC), and cerebellum varies systematically with the direction of reaching movements. These neurons exhibit preferred direction tuning, where the level of neural activity is highest when movements are made in the preferred direction (PD), and gets progressively lower as movements are made at increasing degrees of offset from the PD. Using a functional magnetic resonance imaging adaptation (fMRI-A) paradigm, we show that PD coding does exist in regions of the human motor system that are homologous to those observed in non-human primates. Consistent with predictions of the PD model, we show adaptation (i.e., a lower level) of the blood oxygen level dependent (BOLD) time-course signal in M1, PMC, SMA, and cerebellum when consecutive wrist movements were made in the same direction (0° offset) relative to movements offset by 90° or 180°. The BOLD signal in dorsolateral prefrontal cortex adapted equally in all movement offset conditions, mitigating against the possibility that the present results are the consequence of differential task complexity or attention to action in each movement offset condition.

## Introduction

Neurophysiological studies in non-human primates show that individual neurons in primary motor cortex (M1) [1], [2], premotor cortex (PMC) [3], [4], and cerebellum [5] exhibit preferred direction coding (PDC) whereby the frequency of neural activity is highest in the preferred direction (PD) and progressively reduced as movements are made at increasing angular offsets from the PD. A simplistic model of PDC tuning suggests that the activity of individual cells exhibiting PDC fits a cosine function in which neural activity is greatest for movements in the PD, diminished by half for movements offset by 90° from the PD, and reduced to baseline for movements offset by 180°. It is thought that large populations of these broadly-tuned, direction-sensitive neurons encode the actual direction of the intended movement in a manner analogous to vector averaging [1], [6].

Single-unit evidence for direction coding in human M1 comes from experimental work in paralyzed patients learning to control basic neuroprosthetic devices such as a computer cursor [7], [8]. However, the ability to accurately decode directional information for prosthetic limb control remains problematic [9]. Moreover, given known neuroplastic changes and evidence for abnormal activation patterns in the motor cortex of patients with spinal cord injury [10], PDC may vary between healthy controls and paralyzed patients. Accordingly, a non-invasive technique to measure PDC would provide a valuable opportunity to study the fundamental mechanisms of motor control throughout the intact and diseased nervous system.

In the present investigation, we used functional magnetic resonance imaging adaptation (fMRI-A) [11], [12] to explore the coding of movement direction in the human sensorimotor system. This technique is useful for identifying the nature of information encoded collectively by neurons within a relatively large volume of neural tissue. If the neurons within that volume encode information that is relevant for a specific task, then repeating the task with the same information leads to a reduction in the BOLD signal over time, presumably because the same neurons are engaged repeatedly and adapt over time. In contrast, repeating the task with different information mitigates against reductions in the BOLD signal, presumably because different neurons are recruited [13]. According to this logic, if neurons within a region of motor cortex encode information about movement direction, then repeated movements in the same direction should be associated with adaptation (or “repetition suppression”) of the BOLD signal whereas consecutive movements in disparate directions should be associated with a relative lack of BOLD adaptation. Based on the putative cosine tuning curves described earlier for neurons in M1, PMC, and cerebellum [1], [6], we predicted that maximal BOLD adaptation would be observed when participants made consecutive reaching movements in the same direction (0° offset) relative to consecutive movements offset by 90° or 180°. We further predicted intermediate adaptation of the 90° condition relative to the 0° and 180° conditions.

Note that because adaptation occurs very rapidly (e.g. can begin in less than 100 ms in the visual system) and plateaus within approximately 1 s [13], we expected that adaptation would be observed in the form of an overall reduction of the BOLD signal in the 0° relative to the 90° and 180° conditions throughout the entire movement-block time course. Although block and event-related adaptation designs reveal similar results generally, the use of a blocked design allows an examination of the reliability and consistency of potential adaptation effects over time [13]. While our study is not the first to use fMRI-A to study the human sensorimotor system [12], [14], [15], [16], to our knowledge, it is the first to examine the direction encoding properties of neural populations throughout the motor system with this technique.

## Materials and Methods

The authors declare that all experiments on human subjects were conducted in accordance with the Declaration of Helsinki and that all procedures were carried out with the adequate understanding and written consent of the subjects. The study was approved by the Capital Health Research Ethics Board (Halifax, Nova Scotia).

### Participants

Twelve participants (7 male, 5 female; mean age  = 25) were recruited from the local university community by word of mouth. Participants were eligible to participate in the study if they were right handed, had normal or corrected-to-normal vision, and had never been diagnosed with any psychiatric or neurological disorders, as determined by self-report. All participants received extensive training in the following two Training Tasks before participating in the fMRI-A experiment.

### Training Task I

This task was designed to ensure that participants could maintain visual fixation on a central stimulus while making accurate movements that were similar to those required in the subsequent fMRI-A experiment. The ability to maintain visual fixation during the task was necessary to ensure that measured brain activity was related to movements of the arm rather than the eyes (cf. [17]). Thus, although eye-tracking was not possible during fMRI data-acquisition, the extensive training described below made it unlikely that participants made eye movement during the actual fMRI-A movement task. Movements were made using a joystick handle that was approximately 9 cm in length, 1.3 cm in diameter, and had a maximum excursion of 8°. Participants were seated upright in front of a computer screen at a viewing distance of approximately 58 centimeters. Eye position was monitored using an EyeLink™ II (SR Research, Osgoode, ON) video-based eye-tracking system (monocular sampling rate  = 500 Hz, spatial precision <0.01°, spatial accuracy <0.8° RMS error). Joystick response directions were monitored through a computer interface to evaluate performance accuracy.

Stimuli were similar to those used in the actual fMRI movement task, consisting of a fixation circle (3.15°) surrounded by 8 circular targets (eccentricity  = 4.6°, size  = 2.5°) distributed evenly around an invisible circle in 45° increments. Targets and the fixation circle remained visible throughout the task. The task consisted of discrete trials in which two arrows (1.5° length, 0.5° width) were presented consecutively in the center of the fixation circle with an inter-stimulus interval of 1.5 s. Individual trials were separated by 4 s. Participants were instructed to hold the joystick handle using a power-grasp (i.e. all fingers) while pushing the handle using the forearm and wrist in the direction specified by each arrow as it appeared, quickly and accurately, while maintaining fixation on the central circle. Thus, movements to the left, right, up, and down required supination, pronation, radial, and ulnar deviation respectively. Each arrow could point to any of the 8 target locations, creating trials in which the consecutive joystick movements were offset by a relative angle of 0°, ±45°, ±90°, ±135°, or 180°. Within a block of trials (n = 64), each of the 8 relative offset conditions appeared 8 times in random order, once for each of the 8 possible initial arrow directions.

After each movement, participants were instructed to allow the joystick to return to center by relaxing the wrist muscles. An error message was displayed and the trial was aborted if: the participant took more than 1.5 seconds to respond to the first or second arrow, responded in the wrong direction, or moved their eyes out of the fixation circle during the trial. Participants were required to complete additional blocks of 64 trials until they achieved 95% accuracy in an entire block of trials.

### Training Task II

On the same day as training task 1, participants were trained in a simulated fMRI environment to perform the same reaching task used during fMRI-A data collection (described in the next section). Participants were observed by an experimenter and given feedback to reduce any head, neck, or shoulder movement during the joystick movement task. The simulated fMRI environment consisted of a large acrylic tube with a vinyl-covered foam bed, matching the bore dimensions of the MRI scanner used in the final phase of the study. Participants lay in a supine position with the head inside a smaller acrylic tube matching the dimensions of the head-coil used in the MRI scanner. Similar to the scanning environment, visual stimuli were back-projected onto a small translucent screen mounted to the dorsal end of the simulated head-coil, which participants viewed through a small mirror mounted in front of their eyes. Visual stimuli consisted of a fixation circle and eight peripheral targets as described in training task 1. Via headphones, participants listened to a continuous recording of the noises made during the actual fMRI pulse-sequence while performing this training task. The joystick was positioned in the midline on the abdomen, out of sight from the participant. A failure to respond in the correct direction or a movement response made before the presentation of an arrow resulted in an error. Participants were required to complete runs of trials (described below) until achieving 95% accuracy in an entire run. Participants were given the opportunity to withdraw from the study if they did not feel comfortable in the simulated MRI environment, but no participant withdrew due to this reason.

### fMRI Adaptation Experiment

The experiment was divided into four separate runs, each consisting of 33, 18-second blocks in which participants either made visually-cued joystick movements (16 ‘move’ blocks) or viewed the same visual-cueing sequence without making movements (17 ‘visual-control’ blocks). Each run began with an 18-second instruction screen to allow for scanner warm-up. Each run then commenced with a visual-control block followed by alternating blocks of ‘move’ and ‘visual-control’. Each run lasted a total of 612 seconds (10 minutes and 12 seconds). The importance of maintaining visual fixation and keeping the head, neck, and shoulder as still as possible during scanning was emphasized to participants.

A visual array consisting of a fixation circle and 8 peripheral targets (identical to the stimuli described in training task 1) remained visible throughout all blocks. Although 8 targets were visible, only 4 of these targets, at the cardinal positions (up, right, down, left), were signaled during the task. Visual-control blocks were distinguished from move blocks by the colour of the central fixation circle, which was red or green respectively. Within each 18-second block, a sequence of two consecutive arrows was repeated 6 times (ISI  = 500 ms). Within each sequence of 2 arrows, either arrow could point at any of the four targets (up, right, down, left). As a result, there were 16 unique sequences of arrows (i.e., all possible pairs of the 4 movement directions).

The order of blocked sequences was randomized in each run (with the restriction that a visual-control block always followed a move block). Although there were 16 unique sequences of arrows, the sequences sharing a common offset were collapsed into 4 categories defined by their *relative offset* (0°, 90°clockwise [CW], 90°counter-clockwise [CCW], and 180°). For clarity, all of the sequences and their relative offset categories are shown in Table 1.

**Table 1:** Summary of the movement sequences in each functional run and their corresponding relative Offset conditions.

First Arrow	Second Arrow	Relative Offset Condition
Up	Up	0°
Right	Right	
Down	Down	
Left	Left	
Up	Right	90°CW
Right	Down	
Down	Left	
Left	Up	
Up	Left	90°CCW
Left	Down	
Down	Right	
Right	Up	
Up	Down	180°
Down	Up	
Right	Left	
Left	Right	

### Data Collection

The MRI experiment was completed on the day following the completion of the two training tasks. Scans took place in a 1.5T General Electric MRI scanner. High-resolution anatomical images were collected prior to functional data collection. Full brain T1-weighted spoiled GRASS anatomical images were collected using standard imaging parameters (echo time [TE] = 5 ms; repeat time [TR] = 25 ms; field of view [FOV] = 256 mm×256 mm; 1.5×1×1 mm voxels; 102 sagittal slices). During the 4 functional runs, T2*-weighted, gradient-echo, spiral images (TE = 40 ms, TR = 3 s, flip angle [∝] = 90°) were collected. Functional images were collected of the whole brain using 28 contiguous horizontal slices (FOV = 240 mm×240 mm; in-plane resolution 64×64 pixels) that were each 3.75 mm thick with a 0 mm interslice gap. Average effective voxel sizes were 3.75×3.75×3.75 mm.

### Data Analysis

#### Anatomical Data Processing

Anatomical images were skullstripped with the FSL [18], [19] brain extraction toolkit (BET) (Smith, 2002). Skullstripping involved removal of bone and non-brain tissues from the anatomical images in order to accurately delineate brain tissues from the skull. Images were aligned using an affine transformation to the standard MNI-152_T1 (1 mm^3^) template using the FSL linear registration toolkit (FLIRT) [20] with 12 degrees of freedom (alignment across 3 translations, 3 rotations, 3 scaling factors, and 3 shearing factors).

#### Preprocessing of Functional Data

From the 204 total TRs (time to repetition; the amount of time required to complete scanning of the full region specified, here equaling 3 seconds) for each run, the first 6 were removed to compensate for scanner warm up. The images from the remaining 198 TRs were spatially aligned to a middle (98^th^) time volume. Because a SPIRAL acquisition sequence was used, slice-timing corrections were unnecessary. Results of motion correction were examined visually for excessive motion (any sudden movement which exceeded more than half a voxel over 2 consecutive TRs); TRs with excessive motion were excluded from subsequent analyses. Less than 1% of trials were excluded for this reason; moreover, the movement task did not induce head-movement artifacts (i.e. motion associated with movement of the joystick), indicating that participants were making the joystick movements as trained. Functional volumes were skull stripped (i.e. suprathreshold voxels outside of brain tissue were removed from data analysis) using BET in FSL. Next, functional images were aligned to the subject's anatomical brain images using FLIRT (FSL) with 7 degrees of freedom (alignment across 3 translations, 3 rotations, and 1 global scaling factor). Functional volumes were spatially smoothed with a Gaussian kernel (full-width half-maximum [FWHM] of 6 mm) (3dmerge; AFNI). In order to compensate for minor head movements resulting in a shift of voxels within any particular functional run, a single subject functional mask was made in AFNI (3dautomask) [21] to include only those voxels that overlapped in all 4 runs.

#### Functional Localizer Analysis

Anatomical ROIs were specified *a priori* and located using standard atlases available in FSLview (FSL). The spatial co-ordinates of these ROIs were re-sampled to match the FOV and voxel size used for our functional analyses. ROIs were specified independently for bilateral structures in order to compare activation in the left and right hemispheres. ROIs included M1 (BA -4a and -4p), PMC, supplementary motor area (SMA), and cerebellum. Within each ROI, it was anticipated that many voxels would contain neurons that were not engaged by our specific movement task because of the restricted number of involved muscles. Thus, we carried out the following functional localizer analysis to identify the voxels within the ROIs that were sensitive to the movement task utilized in our experiment; subsequent ROI analyses were restricted to those voxels that were sensitive to the contrast between movement and visual-control conditions.

For the functional localizer analysis, the BOLD signal timecourses from the four functional runs for each subject were concatenated and linear drift was corrected using 3dDeconvolve (AFNI). A reference timecourse was created mapping each TR in the experiment to its associated task condition (0°, 90°CW, 90°CCW, 180°, and visual-control). This timecourse was converted to a vector contrasting the visual-control conditions with the average activity from all movement conditions (i.e. collapsing across the 4 movement conditions), and was convolved with the ideal HRF using the default cox-special WAV form (Waver; AFNI). A one-way repeated measures analysis of variance was calculated for each voxel in the dataset using 3dANOVA2 (AFNI), with task condition as the fixed effect (0°, 90°CW, 90°CCW, 180° versus visual-control) and subjects as the random effect. Statistical correction for false positives was completed using the false discovery rate (FDR) procedure in AFNI [22], ensuring a familywise alpha of 0.05 for the analysis.

#### Region of Interest Analysis

In addition to the sensorimotor ROIs outlined earlier, two more ROIs were created post-hoc to examine the BOLD signal in bilateral dorsolateral prefrontal cortex (DLPFC). Analysis of BOLD timecourses within all ROIs included only those voxels identified in the group functional localizer analysis described above. This voxel-selection procedure was implemented using 3dmaskdump (AFNI). The BOLD signal was calculated for each move condition at each of 10 timepoints (including the 6 TRs associated with the condition plus the preceding 1 and subsequent 3 TRs). BOLD intensity values for each voxel in each condition were normalized relative to the mean activation across all conditions (v_n_  = 100*(v_a_-x)/x), where v_n_  =  normalized voxel intensity, and v_a_  =  absolute voxel intensity) and analyzed for each ROI using separate Movement Offset (0°, 90°CW, 90°CCW, 180°) x Time (1–10 TRs) fully-repeated measures ANOVAs. Only the 6 TRs associated with each movement condition were entered into the ANOVAs.

## Results

### Locating Task-Sensitive Voxels within ROIs

**Figure 1:**
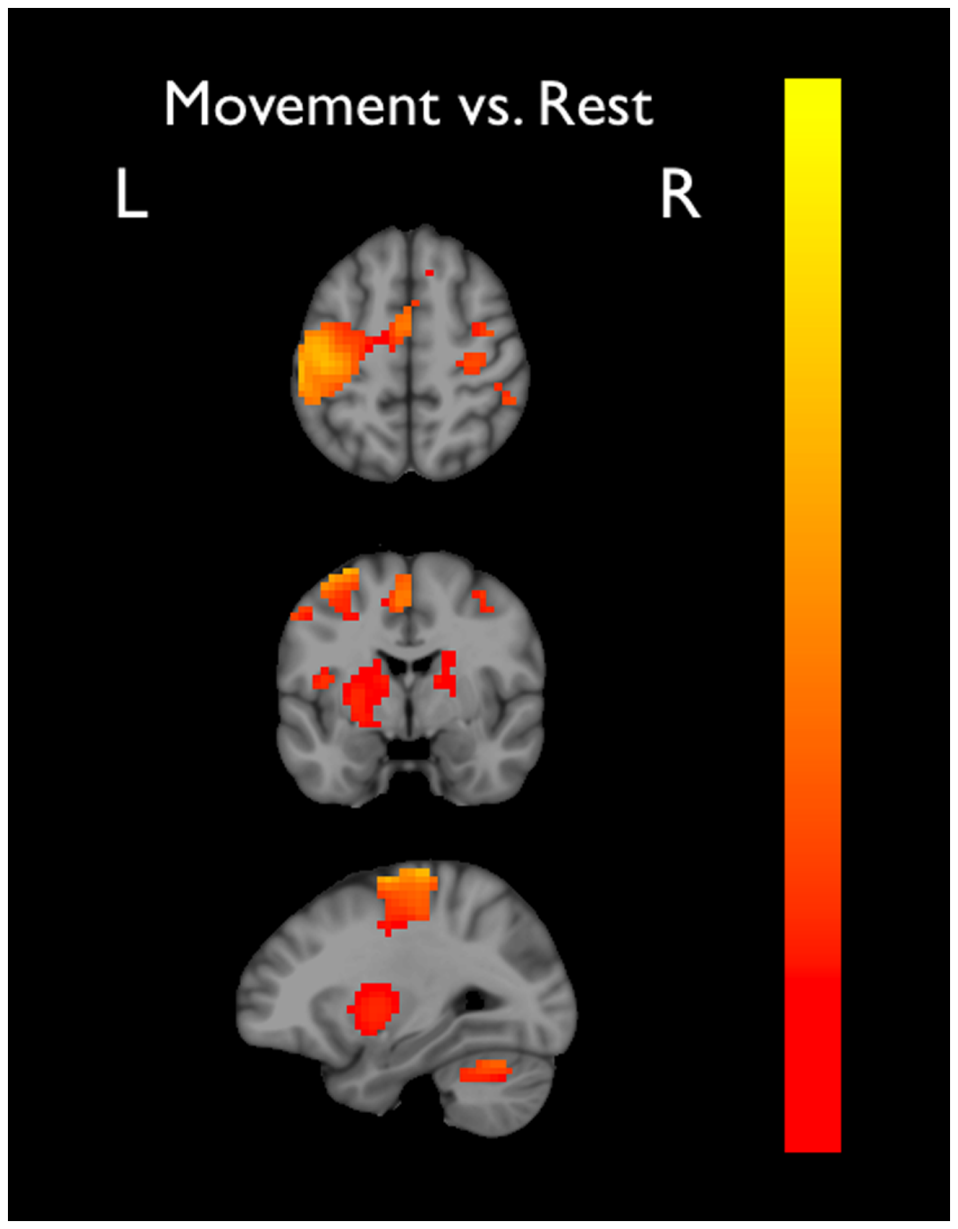
Activation clusters collapsed across all movement conditions compared to the visual-control (baseline) condition in the coronal, axial, and saggital plane at MNI coordinates: 28, 3, 54 (x,y,z). Colour scale represents normalized % signal change (0  =  red; 1  =  yellow).

As expected, the contrast between movement and visual-control conditions identified task-sensitive voxels in bilateral motor cortex (M1, PMC, and SMA; BA -4a, -4p, and -6), cerebellum, somatosensory cortex, insular cortex, inferior parietal cortex, DLPFC, lentiform nucleus, and thalamus (Figure 1).

### ROI Analysis of BOLD Adaptation

**Figure 2:**
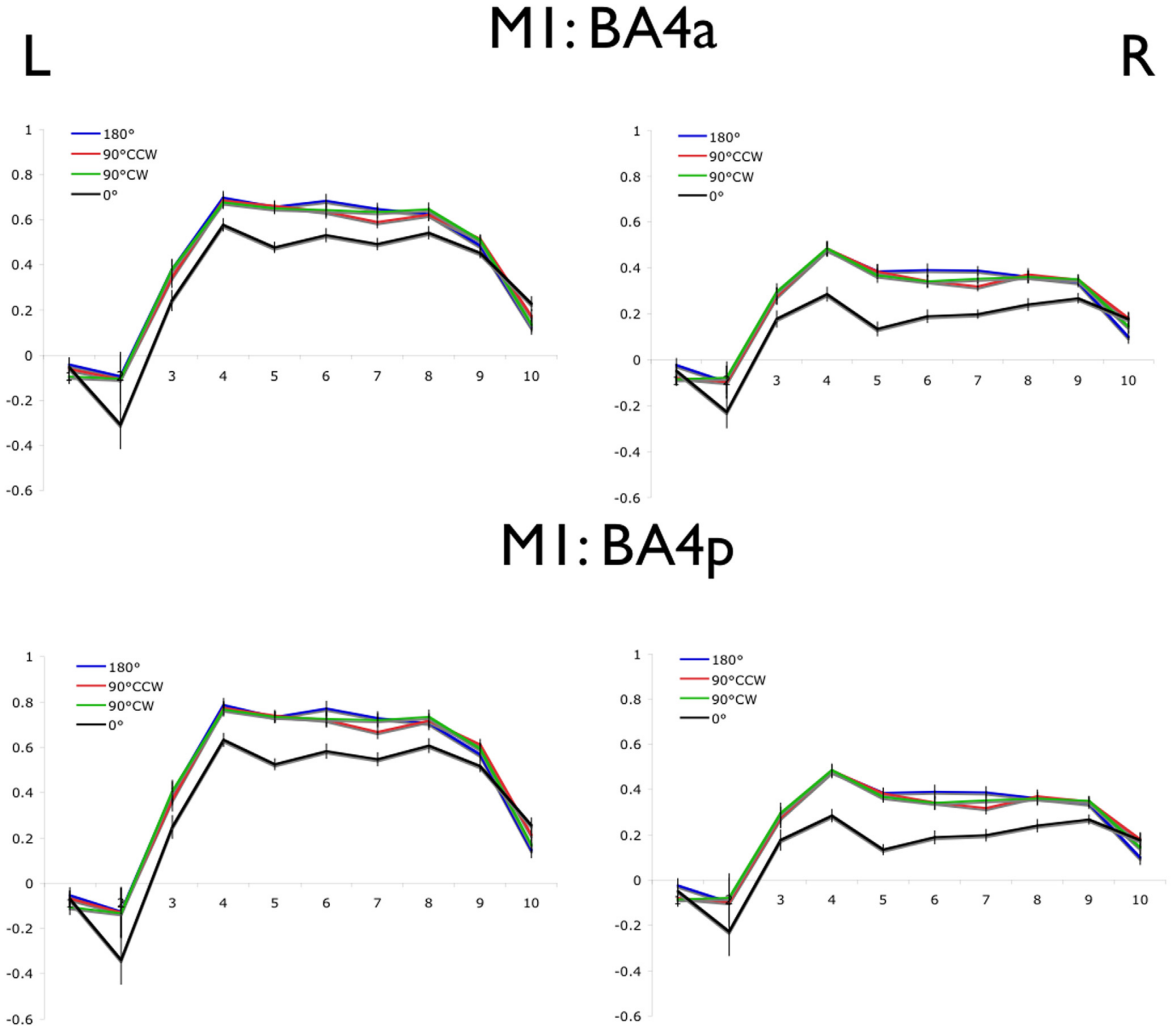
Blood oxygen level dependent signal timecourse in M1 (BA -4a and -4p). Error bars represent the within-subjects standard error at each time point. Time to repetition (TR) is shown on the x-axis and normalized % signal change on the y-axis.

**Figure 3 pone-0013330-g003:**
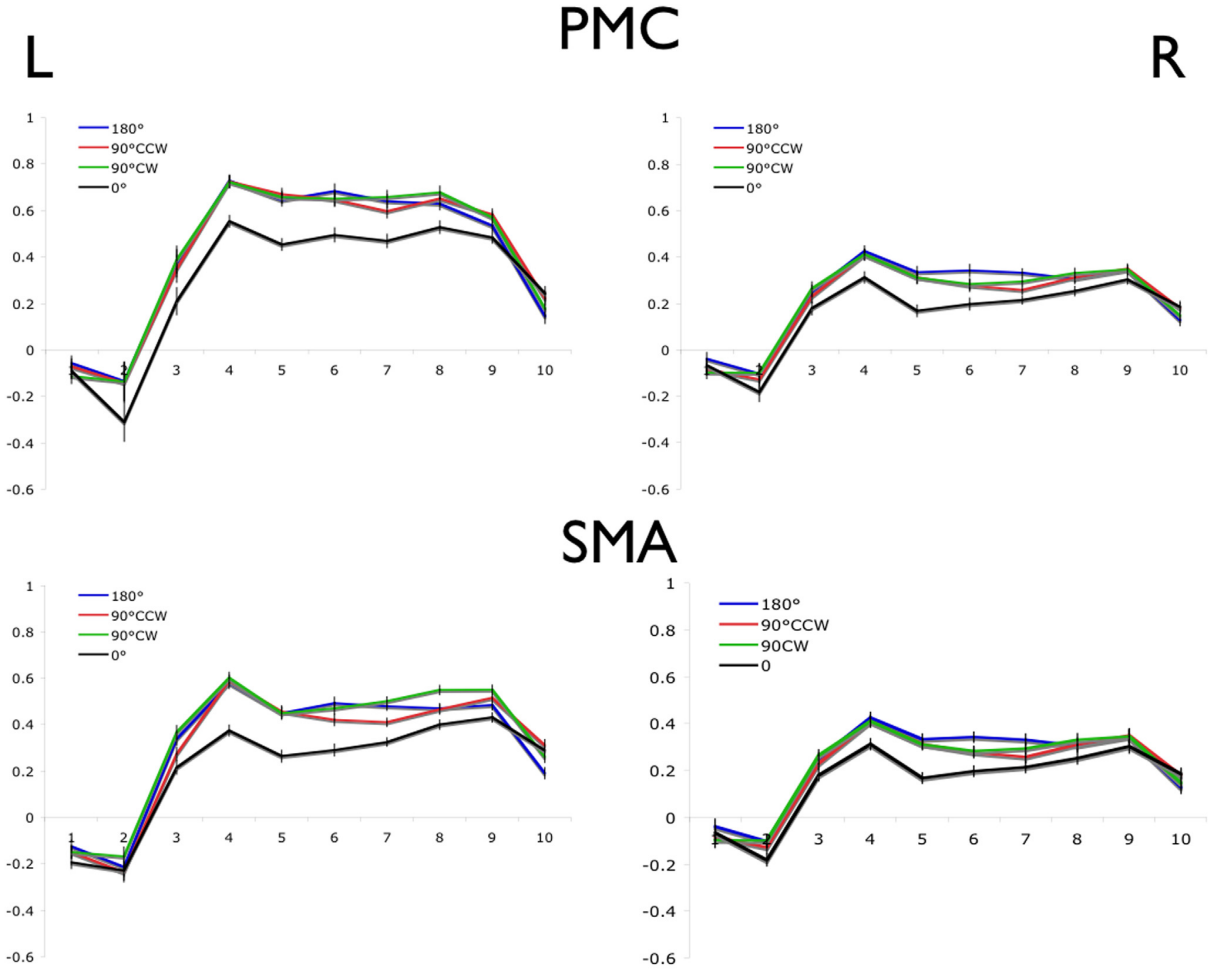
Blood oxygen level dependent timecourse in PMC and SMA (BA6). Error bars represent the within-subjects standard error at each time point. Time to repetition (TR) is shown on the x-axis and normalized % signal change on the y-axis.

**Figure 4:**
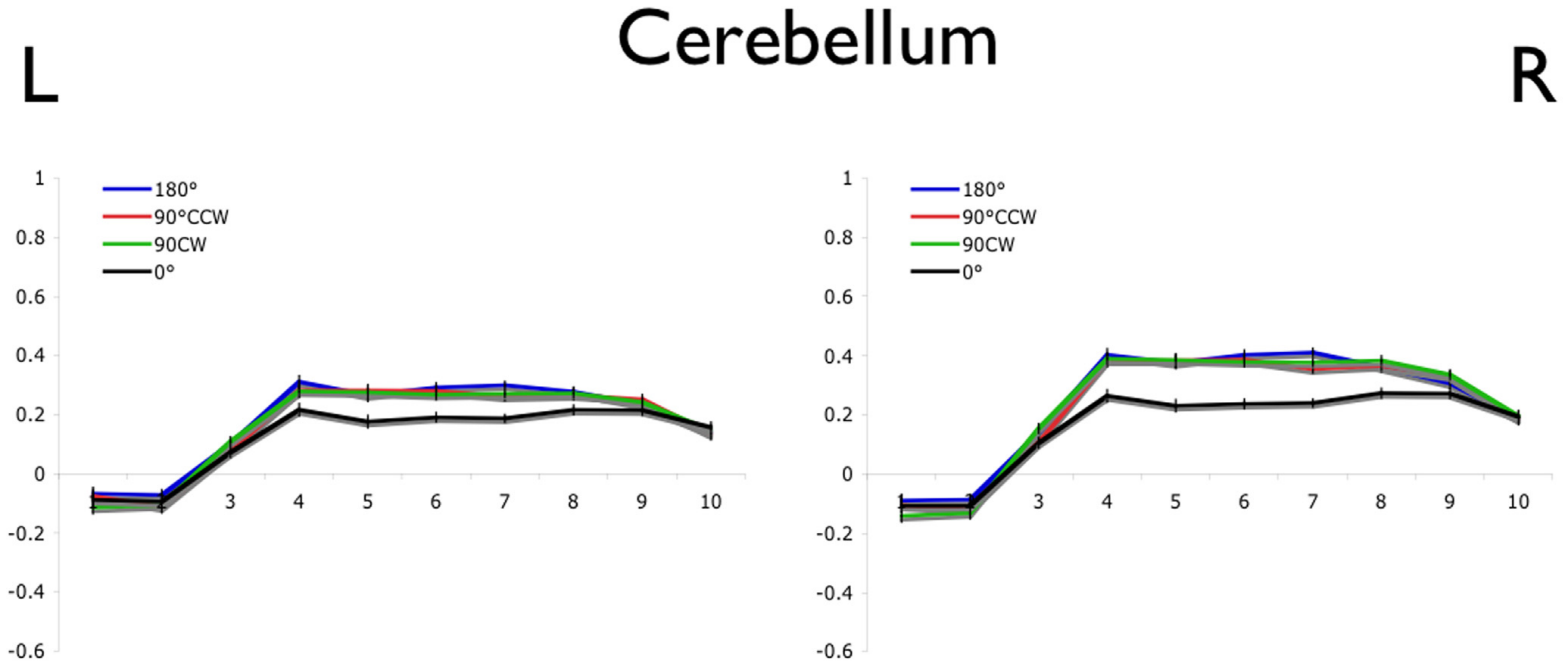
Blood oxygen level dependent timecourse in cerebellum. Error bars represent the within-subject standard error at each time point. Time to repetition (TR) is shown on the x-axis and normalized % signal change on the y-axis.

The average normalized BOLD values are shown for each of the 4 movement conditions in left and right M1 (BA -4a and -4p; Figure 2), PMC and SMA (Figure 3), and cerebellum (Figure 4). In each region, a main effect of movement offset was observed (Table 2). As predicted for each of the bilateral M1, PMC, SMA, and cerebellum ROIs, a lower overall BOLD signal was observed throughout the block time-course in the 0° relative to the 90°CW, 90°CCW and 180° movement conditions. Contrary to predictions, however, no difference was seen between the 90°CW/90°CCW and 180° movement conditions.

**Table 2:** Summary of ANOVA results for the main effect of Offset as a function of brain region.

Brain Region	F(3,33)	p
BA 4a-L	10.64	<.001
BA 4a-R	14.38	<.001
BA 4p-L	8.80	<.001
BA 4p-R	17.40	<.001
PMC-L	10.50	<.001
PMC-R	6.89	<.001
SMA-L	13.11	<.001
SMA-R	10.68	<.001
Cerebellum-L	9.04	<.001
Cerebellum-R	10.25	<.001
DLPFC-L	1.06	.379
DLPFC-R	1.58	.211

**Figure 5:**
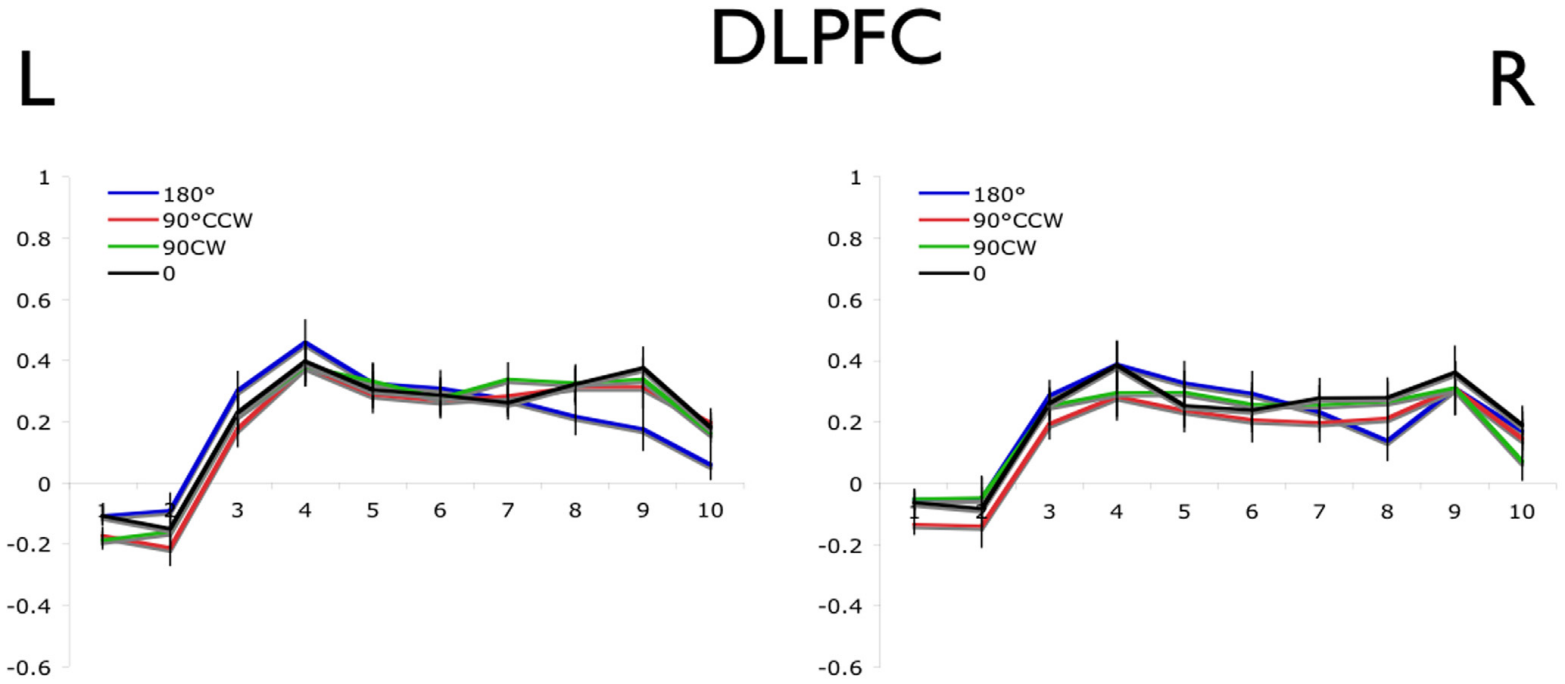
Blood oxygen level dependent timecourse in DLPFC (BA -9 and -44). Error bars represent the within-subject standard error at each time point. Time to repetition (TR) is shown on the x-axis and normalized % signal change on the y-axis.

The greater adaptation of the BOLD signal in M1, PMC, SMA, and cerebellum for the 0° versus all other offset conditions is consistent with the hypothesis that these regions contain populations of neurons with preferred reaching directions. However, a similar result may also be predicted if the attentional or cognitive demands were different for the 0° condition compared to the others. To address this alternative possibility, we examined the BOLD signal in the DLPFC (Figure 5), a region implicated in attentional and cognitive processes (Hoshi and Tanji, 2004; Hoshi, 2006). This analysis revealed a similar BOLD signal in all movement conditions, arguing against a cognitive or attentional account of the data obtained from the other motor regions.

## Discussion

The main finding of the present study is greater adaptation of the BOLD signal in M1, PMC, SMA and cerebellum when participants made repeated movements in the same direction, compared to conditions in which the movements were offset by 90° or 180°. We predicted that adaptation of the BOLD signal would be observed when consecutive movements are made in the same direction due to the repeated activation of the same population of neurons coding for that direction [13]. In contrast, when consecutive movements have different directions, more than 1 subpopulation of neurons should be activated resulting in attenuated BOLD adaptation compared to the 0° condition. These results provide the first non-invasive evidence that healthy human M1, PMC, SMA, and cerebellum encode movement direction in a functionally homologous way to non-human primates, a finding that was very recently reported in M1 [14]. The results of this study both extend those of Eisenberg et. al. (2010), to demonstrate direction encoding throughout the motor system (consistent with neuophysiological studies in non-human primates), and further support the use of fMRI-A as a valuable tool for studying the information encoded by sensorimotor regions of the central nervous system.

Neurophysiological studies of non-human primates suggest that neurons with similar preferred movement directions are organized in a columnar manner, with all possible movement directions represented within approximately 2 mm of cortex [23]. Given the relatively coarse spatial resolution that is associated with whole-brain fMRI, it is not feasible to identify the sub-millimetre topography of direction-encoding units within the human cortex and cerebellum. However, further imaging studies may be able to achieve this goal using alternative techniques such as focusing on specific regions of interest using surface coils to increase signal-to-noise ratio.

Interestingly, significant bilateral activation was observed in each of the motor ROIs specified (Figures 2–4) even though participants only used the right upper limb to perform the task. Although the functional significance of bilateral activation remains unclear, this is not an unusual finding for unilateral movements with either the upper or lower limb [24], [25]. Critically, Horenstein et al. (2009) showed that bilateral activation was not necessarily the consequence of mirroring (i.e. movement of both hands during the unilateral task) by measuring movements of both hands using fiber-optic gloves.

### Precision of Directional Tuning Curves

Contrary to our prediction, we found similar BOLD adaptation in the 90° conditions relative to the 180° condition. We predicted greater adaptation of the BOLD response in the 90° conditions relative to the 180° condition based on the assumption that neurons exhibiting PDC have tuning curves that fit a cosine function [1], [2], [4], [6] wherein the neuron fires at half of its maximal rate when a movement is made 90° from the neuron's PD, and activity drops to baseline levels for movements made 180° from the PD. If this were the case, then the subpopulations of neurons engaged during consecutive movements offset by 90° should overlap to a large extent, causing a level of BOLD adaptation intermediate between that observed in the 0° and 180° conditions.

Although the vast majority of studies describe PD tuning with a cosine function, a comprehensive examination of direction encoding across 20 movement directions (rather than the 8 directions typically used in earlier papers presenting the cosine model), reveals that the tuning profiles of most neurons in non-human primate motor cortex exhibit tuning curves that are significantly narrower than that described by a cosine wave form [26]. Specifically, neurons in the monkey motor cortex exhibit tuning curves with a half-maximal width that varies between 30° to 90° with a mode of approximately 50°. In fact, less than 17% of neurons exhibited tuning curves with a half-maximal width greater than 80°. Therefore, in contrast with our original predictions, it seems likely that movements offset by 90° are controlled mostly by distinct rather than overlapping populations of neurons. A similar conclusion was reached by Eisenberg et al. (2010), who did not observe fMRI adaptation in human M1 for repeated movements offset by 45–180°. Eisenberg et al. suggest that tuning curves may be narrower in humans compared with monkeys in a variety of brain areas ranging from primary auditory cortex [27] to M1. If the preferred direction tuning curves in human sensorimotor areas are indeed narrower than a cosine function, then repetition supression may only be observed when repeated movements are offset by less than 45°, not 90° as we had originally predicted.

### Task Complexity

One alternative hypothesis for the difference in repetition suppression between the movement offset conditions is that repeated movements in the 0° condition are less complex and require less planning by the motor system compared to all other conditions. If this is the case, then one would expect to see the same adaptation pattern in areas responsible for strategic planning of motor actions, such as the dorsolateral prefrontal cortex (DLPFC). The DLPFC appears to play a central, integrative role in the modulation of motor control by selecting action strategies based on multiple sources of information that are appropriate to the task requirements [28], [29]. Single unit studies in non-human primates and fMRI studies in humans show increased activity of the DLPFC when task complexity is increased [30], [31]. Given that the BOLD signal for suprathreshold DLPFC voxels did not differ between the 180°, 90°CW, 90°CCW or 0° conditions (Figure 5), it is unlikely that task complexity differed between movement conditions.

### Attention

A second alternative hypothesis for the activation differences observed between movement conditions is that the repeated movements in the 0° condition require less specific attention to action than all other conditions. A previous study showed that BOLD activation decreases in area BA4p but remains stable in BA4a when participants decrease attention to their finger movements, suggesting that attending to action does not modulate neural activity in BA4a [32]. Furthermore, the PFC is also known to modulate attention to action in humans [33]. Given that adaptation of the BOLD signal was observed in BA4a in the current study, it is unlikely that attentional variables alone can account for the difference in BOLD activation between movement conditions. Moreover, BOLD signals in the DLPFC did not differ between movement conditions (Figure 5) suggesting that if attention to action was similar across all movement conditions.

### Muscle Activity

An intuitive objection to the present results (and the direction-encoding model in general) is that direction of the movement and the muscle groups required to implement it are confounded. This makes it difficult to conclude whether brain regions encode movement direction per se or merely specific muscle groups. In other words, because the initial orientation of the hand did not change between the first and second movement in a sequence, a relative offset of 0° between movements not only requires a movement in the same direction but also engages exactly the same muscles. Of course, in the 90° and 180° conditions, the directions are different between the first and second movement and distinct groups of muscles are required. Therefore, reduction of the BOLD signal could be a consequence of repeated activations of the same muscle groups in the 0° condition relative to the 90° and 180° conditions, rather than repeated activation of neurons that encode the same intended movement direction. Using wrist movements in monkeys, Strick and colleagues have carefully assessed this potential problem in both M1 [2] and PMC [4]. The majority of neurons in M1 and PMC maintained their PDC encoding despite varying initial wrist posture. In fact, of the neurons showing direction tuning in the PMC, ∼94% showed no shift in their PDC despite large changes in starting wrist posture, whereas the other 6% showed some posture-dependent change. As such, the results of the present study can be attributed with considerable confidence to direction-encoding properties of neurons rather than their connections to specific muscle groups.

### Future Research Directions

Although the sensorimotor areas of the human central nervous system have been extensively mapped, the information processing operations carried out in these regions are not well understood. Specifically, while the motor cortex is thought to simultaneously encode many other movement parameters such as force, speed, acceleration, amplitude, joint rotation, and hand position, the relative encoding and importance of each variable remains unclear. It is noteworthy that whereas changes in force, speed, acceleration, and position are correlated with cellular activity in M1, PMC, and SMA, the correlation is typically strongest with movement direction. Indeed, whereas overall cellular activity is “gain modulated” by various kinematic parameters, the underlying PD tuning curve remains preserved [34], [35], [36]. It is unlikely that movement parameters such as force, speed, acceleration or amplitude can entirely account for the present results because the joystick device limited the range of motion for all movement directions; consequently, movement parameters other than direction were similar across all movements. In other words, two movements in the same direction (i.e., a 0° offset) or two movements in different directions (e.g., a 90° or 180° offset) shared most movement parameters (other than direction), leaving direction as the single factor to account for the repetition suppression observed. That said, the fMRI-A technique may provide a useful tool for further non-invasive studies focused on the neural encoding of other, non-directional movement variables in motor regions of the brain. For example, one could search for regions of motor cortex that encode force by comparing the BOLD response for repeated movements in the same direction between conditions where the level of resistance is held constant or varied between repetitions. The fMRI-A technique may also be implemented using an event-related design in order to assess, in greater detail, the time-course of repetition suppression effects in the motor system. Finally, the fMRI-A technique utilized in the present investigation may be useful in understanding sensorimotor learning and re-learning following neurological injuries such as stroke.
